# Agarose gel electrophoresis of joint fluid using Hyrys-Hydrasys SEBIA system as a new prognostic tool for periprosthetic osteolysisin revision arthroplasty

**Published:** 2013-09-25

**Authors:** A Chiva

**Affiliations:** * Department of Clinical Chemistry-Electrophoretic Laboratory University Emergency Hospital Bucharest

**Keywords:** agarose gel electrophoresis, joint fluid, proteins, wear mediated osteolysis, revision arthroplasty

## Abstract

**Rationale.** Prevention of wear-mediated osteolysis, the most common complication in total joint arthroplasty, is a great challenge for orthopedic surgery. Despite the diversity of current biomarkers of periprosthetic osteolysis (products of wear, bone turnover and inflammatory biomarkers), the major interferences and the great amount of sample necessary for analysis limit their use in clinical practice.

**Objective. **The aim of this paper is to present three new electrophoretic methods using Hyrys-Hydrasys SEBIA system that have been used for the first time in Electrophoresis Laboratory of our hospital in the analysis of joint fluid for the prevention of periprosthetic osteolysis in revision arthroplasty.

**Methods and results.** Analytical aspects of agarose gel electrophoresis of joint fluid proteins and lipoproteins as well as SDS-agarose gel electrophoresis of joint fluid proteins, their performances and clinical value are presented. The decreased level of albumin and increased level of alpha1 and alpha2 globulins were frequent changes detected on SEBIA electropherograms and good indicator for the presence of an inflammatory reaction generated by particle debris. In addition, a slightly increase of LDL mobility could provide good information about a high oxidative stress. Moreover, the Ig G assessed by using SDS-agarose gel electrophoresis could be a potential biomarker for an immunological reaction towards orthopedic implants.

**Discussion.** Electrophoresis of joint fluid using Hyrys-Hydrasys SEBIA France system is a new analytical technique able to remove the most of current biomarkers disadvantages due to the determination of particular proteins (acute phase proteins, albumin, lipoproteins, and immunoglobulins) by using minimal amounts of joint fluid with minor interferences, minimal cost and rapid results.

** Abbreviations** CTX, crosslinked C-telopeptide; IL- interleukins; Ig G, immunoglobulin G; LDL, low density lipoprotein; NTX, crosslinked N-telopeptide; PICP, procollagen I C – terminal extension peptide; SDS, sodium dodecyl sulphate

## Introduction

Wear mediated osteolysis and consequent aseptic loosening is the most common cause of revision of total joint arthroplasty. The chronic inflammation induced by wear debris, macrophage activation and osteoclast mediated bone resorption are the main contributors to periprosthetic osteolysis [**1,2**]. Oxidative and nitrosative stress [**3**] as well as type IV hypersensitivity and other immunological mechanisms [**4**] are important co-players in this process. According to this etiology, current biomarkers of implant wear include markers of bone turnover (propeptide and telopeptide of collagen CTX, NTX, PICP, deoxypyridinoline) [**1,2**] and inflammatory reactions (interleukins, metalloproteinases) [**1,3**] as well as products of wear process (metal ions derived from implants) [**4,5**]. Despite their specificity and sensitivity, the multitude of interferences (age and gender, ethnicity, circadian rhythm, seasonal variation, fractures, immobility, physical activity, menstrual cycle, drug interferences, diet, alcohol consumption, smoking) [**6**] and the less availability of analytical procedure, limit their use as routine tests. The analysis of particular proteins (acute phase proteins, enzymes, immunoglobulins, complement fractions) in synovial fluid from a specific joint is of great interest because it could provide information about the biochemical changes from that specific joint. However, the great amounts of joint fluid necessary for the analysis of every protein mentioned, the viscosity, and the invasive character of arthrocentesis could limit their clinical value. Thus, the identification of the new analytical procedures with minor interferences, which require minimal amounts of joint fluid, is a great challenge in the prevention of orthopedic implant failure.

### Electrophoresis of joint fluid- advantages, methods, performances

 Electrophoresis of joint fluid in Hyrys-Hydrasys SEBIA France system could be a new prognostic tool for wear mediated osteolysis due to its advantages: the qualitative and quantitative analysis of a large number of protein fractions that may be identified by using a small quantity of joint fluid (0.3-0.5 ml), the minor interferences, and the possibility of fast introduction as routine test because of the procedure and instrument availability [**7**]. The invasivity of arthrocentesis has been reduced by the sample collection only intraoperatively and before the incision of joint capsule, in accordance with the guidelines from scientific literature [**8**].

 The first step in electrophoretic analysis of joint fluid is agarose gel electrophoresis, a classic technique that is able to separate 5 or 6 protein fractions: albumin, alpha1, alpha2, beta (total or beta1, beta2) and gamma globulins [**7**]. If some qualitative and/or quantitative abnormalities are detected, other electrophoretic analyses are required in order to extent the number of proteins with clinical value. Thus, joint fluid lipoproteins electrophoresis could be an additional tool for synovial lipoproteins profile and screening of oxidized LDL (low-density lipoprotein), biomarker strongly correlated with the presence and severity of inflammatory process and oxidative stress. In addition, SDS agarose gel can be a useful test for the identification of Ig G, albumin, haptoglobins, and other inflammatory, oxidative stress and immunological markers [**7**]. An association of these types of electrophoresis could considerably improve the clinical value, in particular in special cases (patients with rheumatoid arthritis and osteoporosis who underwent arthroplasty).

 Sample preparation and storage

 The joint fluid samples harvested by arthrocentesis at revision surgery were centrifuged at 2000x g for 10 minutes at room temperature in order to remove cells and other debris. The supernatants aliquoted can be stored until 2 month at - 20ºC [**9**]. For electrophoresis of proteins and lipoproteins, the viscosity of joint fluid was reduced by using Fluidil SEBIA (25μl Fluidil and 75μl joint fluid), a solution frequently used for dilution of viscous or turbid sera. For SDS-agarose gel, a dilution with saline solution is necessary in order to obtain about 0.2 g/dL proteins. No pre-treatment with Fluidil is necessary for this type of electrophoresis.

 Procedures

 All types of electrophoresis were performed by using Hyrys-Hydrasys SEBIA France automated system. Agarose gel electrophoresis was assayed by using Hydragel β1-β2 SEBIA France kit in order to obtain 6 protein fractions (albumin, alpha1, alpha2, beta1, beta2, and gamma globulins) by using an alkaline buffered pH 8.6 agarose gel. The separated proteins were stained with amido black. 

 Electrophoresis of joint fluid lipoproteins was assayed by using Hydragel Lipo+Lp(a) SEBIA France kit which uses a buffered pH 7.5 agarose gel as electrophoretic support. The separated lipoproteins were stained with Sudan black.

 SDS-agarose gel electrophoresis of joint fluid proteins was assayed by using Hydragel Proteinurie SEBIA France kit, a neutral buffered SDS-agarose gel. The electropherograms were stained with acid violet, a stain with a high affinity for weak fractions and hypoproteic biological fluids. 

 Although these kits are intended for separation of proteins in serum and urine, they can be also used for joint fluid analysis because the total proteins level of this fluid (1.5-4 g/dL) is above the limit of detection reported by the producer (1.5 g/dL for Hydragel β1-β2 and Hydragel Lipo and 0.2 g/dL for Hydragel Proteinurie). 

 The programs of migration and gel processing are similar with those for serum and urine, being processed automatically by the instrument in the following sequence: sample application, electrophoretic migration, drying, staining, distaining, and final drying. The electropherograms are evaluated visually for pattern abnormalities. Densitometry provides accurate relative quantification of individual fractions. An assayed control serum SEBIA (for proteins) for an accurate quantification was used into each run of samples. For SDS-agarose gel, a molecular weight control SEBIA was used in order to facilitate a correct identification of the most important proteins: albumin (66 kDa), Ig G (150 kDa), and lysozyme (14.5 kDa).

Performances

The study of analytical performances has demonstrated a good reproducibility within and between run for each type of joint fluid electrophoresis. The lowest concentration of the most important protein fractions that can be detected by classic agarose gel electrophoresis is of 0.02 g/dL and 1.5 mg/dL for SDS-agarose gel electrophoresis (unpublished data). 

 A significant correlation between joint fluid albumin levels measured by using agarose gel and SDS-agarose gel electrophoresis has been demonstrated (r = 0.85) (unpublished data). This is a strong evidence of the accuracy of these methods.

 The reference values were established by using a lot of patients with aseptic loosening due to the mechanical stress (which did not involve biochemical and cellular response to implant debris) (**Table 1**).

**Table 1:** Different types of electrophoresis for joint fluid analysis; reference ranges for the most important biomarkers

Electrophoresis type	Fractions	Reference ranges (%)
Agarose gel electrophoresis	Albumin	54.8 - 60
	Alfa1 globulins	2.3 -3.7
	Alfa2 globulins	8.5 - 9.1
	Beta 1 globulins	5 -7
	Beta 2 globulins	8.3 - 9
	Gama globulins	14 -16.8
Electrophoresis of lipoproteins	Oxidized LDL	46 - 55
SDS-agarose gel electrophoresis	Ig G	10 -14

### Electrophoretic changes of joint fluid proteins in wear mediated osteolysis and aseptic loosening

Different types of electrophoresis can separate many proteins by using Hyrys-Hydrasys SEBIA system (acute phase proteins, albumin, immunoglobulins, LDL / oxidized LDL). Due their role in wear-mediated osteolysis, it is expected that these proteins could be important biomarkers for inflammatory response to particle debris, oxidative stress and immune reactions as important parts of aseptic loosening mechanisms [**7**]. 

Agarose gel electrophoresis of joint fluid proteins

**Fig. 1:**
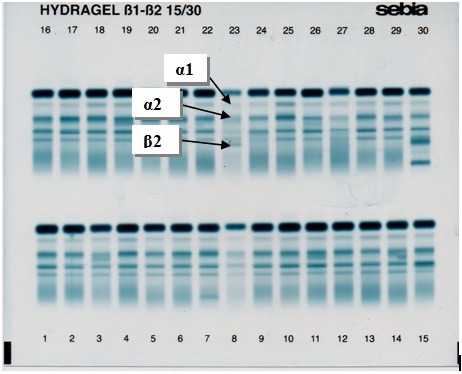
Agarose gel electrophoresis of joint fluid proteins. Alpha 1 and alpha 2 globulins (acute phase proteins) as well as beta 2 globulins, the most important biomarkers for an inflammatory reaction to particle debris are indicated by the arrows

 Acute phase proteins (α1 antitrypsin and α1 antichymotrypsin in alpha1 zone, haptoglobins in alpha2 zone, and complement factor in beta zone) are the most important proteins that can be separated as heterogeneous groups (**Fig. 1**). 

Decreased level of albumin and increased level of alpha1 and alpha2 globulins were frequent changes detected on SEBIA electropherograms [**7**]. The same results have been reported while using other electrophoretic supports [**10**]. Such biochemical changes could provide good information about inflammatory reactions to particle debris and, indirectly, for the reactivity towards implants [**7**]. In some cases, elevated beta 2 globulins levels have been reported, probably due to changes in complement C3 concentration, the major protein of beta zone. Although some recent studies have documented the role of complements in particle mediated recruitment, proliferation, and activation of macrophages during the early events of periprosthetic osteolysis [**11**], the role of beta globulins as biomarkers in the implant failure should be evaluated by further studies.

Electrophoresis of joint fluid lipoproteins

**Fig. 2:**
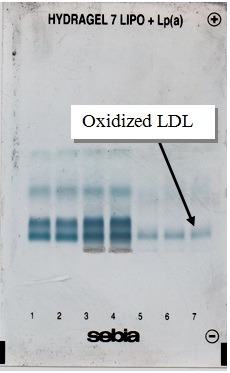
Electrophoresis of joint fluid lipoproteins. In the presence of particle debris and consequent pro-oxidative environment, LDL is middle oxidized with a very slightly increased mobility on electropherograms (position no. 5-7). The comparison was made with the mobility of serum non-oxidized LDL (position no. 1-4)

 The quantification of LDL/oxidized LDL is the main advantage of this electrophoresis (**Fig. 2**). As a result of periprosthetic inflammation, LDL is middle oxidized in joint space. A slightly increase of LDL mobility on SEBIA electropherograms are in accordance with other previous studies [**12**], and a good indicator for a high oxidative stress [**7**].

SDS- agarose gel of joint fluid proteins

**Fig. 3:**
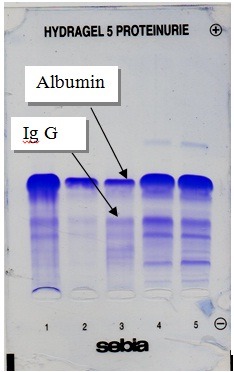
SDS agarose gel electrophoresis of joint fluid proteins. Albumin and immunoglobulins are the most important proteins in joint fluid, providing good information about the presence and severity of the inflammatory process (albumin) or immunological reactions (Ig G) towards orthopedic implants.

 SDS- agarose gel electrophoresis of joint fluid proteins is a very useful additional tool for some biomarkers that cannot be separated using other types of SEBIA electrophoresis (Ig G, haptoglobins, lysozyme, lactotransferrin) [**7**]. Ig G can be measured with good accuracy as a potential biomarker for the hypersensitivity and immune reactions towards the orthopedic implants [**7**] as well as for local immunity capability, usually decreased in a second prosthetic failure due, in particular, to modulation of IL-10 expression [**9**] (**Fig. 3**). 

No significant correlation between the Ig G level in joint fluid from wear mediated osteolysis and those from implant failure due to mechanical stress (considered as reference) has been reported (unpublished data). However, further studies are necessary in order to demonstrate the predictive role of Ig G levels for immunological intolerance of prosthesis (in particular in metal-on-metal implants).

 No previous studies about the predictive value of lactoferrin and lysozyme for aseptic loosening have been published. Taking into account their role in inflammatory reactions due to metal debris, oxidative stress and antimicrobial defense [**13**], it would be expected that these biomarkers could provide information about the presence of a local inflammatory response, as well as antioxidant and antimicrobial defense capacity [**7**]. However, the main limit is that lactoferrin cannot be separated from transferrin (both with a molecular weight of 80 kDa) and measured individually. In addition, the level of these two markers could be often low or non-titratable. Further long-term studies should evaluate their role in the assessment of individual reactivity towards orthopedic implants and risk of prosthetic failure.

## Discussion

Electrophoresis of joint fluid proteins using automated system Hyrys-Hydrasys SEBIA France is a new analytical technique that has been used for the first time in Electrophoretic Laboratory of our hospital [**7**]. No data about the previous use of electrophoresis of joint fluid as routine analysis has been published in scientific literature.

 The association of different types of SEBIA electrophoresis for joint fluid analysis appears to be a viable and robust alternative for the prognostic of periprosthetic osteolysis, with a high potential of fast introducing as routine test. The evaluation of many proteins in joint fluid (acute phase proteins, LDL/oxidized LDL, albumin, immunoglobulins) using a small quantity of sample with minor interferences provide many valuable information concerning the inflammatory response to particles debris, oxidative stress and immunologic reactivity towards implants as important mechanisms of periprosthetic osteolysis [**7**]. Moreover, the presence of some discordances between the severity of wear mediated osteolysis assessed by electrophoresis and radiological techniques is a strong evidence that electrophoresis could play a key role in the prevention of aseptic loosening in patients undergoing joint arthroplasty [**14**].

Although the sample collection is limited to the revision surgery, the role of joint fluid electrophoresis is considerably increased because it offers some information about the biochemical changes that have occurred during the primary implant, thus preventing the risk of aseptic loosening by choosing a suitable implant material, type of prosthesis and further therapy, in particular with antioxidant agents [**3**]. Because the electrophoresis of joint fluid in SEBIA France system has been used for only 3 years and the aseptic loosening could appear in a longer period, the predictive value of electrophoresis should be investigated by further studies.

 In conclusion, despite the main role of these proteins in periprosthetic osteolysis, none of these has absolute clinical value. For an accurate prognosis of aseptic loosening, the electrophoresis results must be correlated with the other biochemical markers and radiological data [**7**]. 
